# Adjunct Intravitreous Triamcinolone Acetonide in the Treatment of Diabetic Macular Edema with Anti-VEGF Agents

**DOI:** 10.1155/2016/5282470

**Published:** 2016-11-17

**Authors:** Robert B. Garoon, Robert E. Coffee, Lai Jiang, Christina Y. Weng, Petros E. Carvounis

**Affiliations:** ^1^Michael E. DeBakey Veterans Affairs Medical Center, Houston, TX, USA; ^2^Cullen Eye Institute, Baylor College of Medicine, Houston, TX, USA; ^3^School of Medicine, Baylor College of Medicine, Houston, TX, USA

## Abstract

*Aims.* To compare visual and anatomic outcomes of adjunct intravitreous (IVT) triamcinolone acetonide to antivascular endothelial growth factor (VEGF) injections to IVT anti-VEGF injections alone for center-involving diabetic macular edema (DME) in treatment-naïve eyes.* Methods.* Retrospective study of treatment-naïve eyes with center-involving DME. The primary outcome was the change in best corrected visual acuity (BCVA) in eyes receiving only IVT anti-VEGF (group 1) and eyes receiving IVT anti-VEGF and adjunct IVT-TA (group 2).* Results.* Included were 192 eyes. The mean change in BCVA was +3.5 letters in group 1 compared to −3.5 letters in group 2 (*p* = 0.048). Final macular thickness improved by −94 *μ*m in group 1 versus −68 *μ*m in group 2 (*p* = 0.26). In group 1, 5/150 eyes compared to 9/42 eyes in group 2 (3.3% versus 21%, *p* = 0.0005) had a IOP >10 mmHg increase. Six of 126 phakic eyes in group 1 versus 12/33 phakic eyes in group 2 underwent cataract surgery (4.7% versus 36.3%, *p* = 0.00009).* Conclusions.* IVT-TA results in no additional benefit in eyes treated with anti-VEGF agents for DME.

## 1. Introduction

Diabetic macular edema (DME) is the leading cause of moderate vision loss in patients with type 2 diabetes mellitus [1, 2]. Control of hyperglycemia, hypertension, and dyslipidemia has been associated with reduced risk of development of diabetic macular edema and visual loss [3–7]. Macular LASER photocoagulation (MLP) was shown to effectively treat DME in the Early Treatment Diabetic Retinopathy Study (ETDRS) [8]. More recently, large, well-conducted randomized controlled studies have shown that treatment with intravitreous (IVT) antivascular endothelial growth factor (VEGF) agents such as bevacizumab, ranibizumab, and aflibercept for DME results in superior visual and anatomic outcomes compared to macular LASER photocoagulation [9–16]. Patients with visual acuity worse than 20/40 at baseline were recently shown to have superior visual outcomes when treated with IVT aflibercept compared to the other 2 anti-VEGF agents [17].

Intravitreous triamcinolone acetonide (TA) has been used for treating DME since 2001 [18]. A large, randomized control study showed similar visual outcomes following treatment of DME with IVT-TA compared to MLP; however IVT-TA resulted in progression of cataracts in half of phakic eyes and ocular hypertension in a third of eyes [19]. For pseudophakic eyes, a large, randomized controlled study demonstrated that mean visual gain was similar between eyes treated with IVT-TA with MLP and IVT-ranibizumab [13]. Moreover, TA improved vision in cases of DME refractory to bevacizumab, with anatomic efficacy predicted by high intraocular IL-8 levels [20].

Anti-VEGF medications and corticosteroids are capable of inhibiting different cytokine-mediated cascades to decrease the amount of macular edema, suggesting that there may be benefit in combining treatments [21–24]. Smaller studies have failed to show benefit of adjunct IVT-TA to anti-VEGF for the treatment of DME [25–27].

We undertook the present study in order to determine whether use of adjunct IVT-TA in combination with IVT anti-VEGF resulted in improved visual and anatomic outcomes compared to IVT anti-VEGF alone in a clinical setting beyond the short term.

## 2. Materials and Methods

This study was a retrospective chart review of consecutive patients who had been examined by either or both of two retina specialists in the retina clinic at the Michael E. DeBakey Veterans Affairs Medical Center (MEDVAMC) from January 2012 to November 2014. The study was approved by the Institutional Review Boards of both Baylor College of Medicine and the MEDVAMC and complied with Health Insurance Portability and Accountability Act regulations.

Patients with a diagnosis of center-involving diabetic macular edema who received at least 3 intravitreous injections over the course of 6 months and had total follow-up of at least 12 months were included. Exclusion criteria were concurrent macular pathology other than diabetic retinopathy, follow-up of less than 12 months and treatment with macular laser photocoagulation, IVT anti-VEGF, or IVT-TA injection prior to enrollment in this review. Subjects were analyzed from the initiation of therapy until the end of the chart review period.

Patients were asked to return every 4 weeks and electronic ETDRS best corrected visual acuity (BCVA) was documented by ophthalmic technicians at each visit. Spectral-domain optical coherence tomography (SD- OCT) (Spectralis, Heidelberg Engineering, Heidelberg, Germany) was obtained and central subfield macular thickness (CSMT) was determined using the fast-scan protocol. The decision to administer an intravitreous injection was made by a retinal specialist if there was intraretinal fluid on SD-OCT and BCVA less than 20/20 or if diabetic macular edema was adjudged to be distorting normal foveal architecture; otherwise the patient was observed. A decision to switch anti-VEGF agent was made by the retina specialist when there had been worsening or no improvement (typically <5–10% change) of CSMT on SD-OCT at two consecutive visits. One of the two attending retina specialists routinely used adjunct triamcinolone acetonide in combination with anti-VEGF treatment when resolution of intraretinal fluid after a previous IVT anti-VEGF injection had been incomplete or when he judged that the diabetic macular edema was severe as defined by CSMT greater than 365 *μ*m (30% greater than normal retinal thickness).

Eyes were divided into two main groups: group 1 eyes underwent treatment for DME with anti-VEGF agents only, including bevacizumab (Avastin, Genentech, San Francisco, CA), ranibizumab (Lucentis, Genentech, San Francisco, CA), or aflibercept (Eylea, Regeneron, New York, NY), while group 2 eyes received 2 mg of IVT preserved triamcinolone acetate (Kenalog, Bristol-Myers-Squibb, Princeton, NJ) in some of their visits while receiving anti-VEGF agent treatments. The main outcome measure was the difference in mean BCVA between the two groups at 1 year. Secondary outcome measures included the difference in BCVA between the two groups at final follow-up, the change in CSMT at 1 year and at final follow-up, the proportion of patients who underwent cataract extraction, the change in mean IOP at final follow-up, the proportion of patients that had an increase in IOP >10 mmHg from baseline, the proportion of patients that had IOP-lowering medication initiated at any visit, and the number of patients needing glaucoma surgery at any time during follow-up. A subgroup analysis of eyes that had DME refractory to anti-VEGF treatment (less than 10% improvement from baseline CSMT after 3 or more injections of IVT anti-VEGF medication) and eyes that received an early IVT steroid injection before receiving 3 injections of IVT anti-VEGF medications was also performed: the former was group 2A and the latter was group 2B.

For the purposes of statistical analysis, BCVA visual acuities were converted to logarithm of the minimum angle of resolution (logMAR). Patients with count finger visual acuities were assigned a logMAR value of 1.6. Independent *t*-test and ANOVA statistical analysis were used to compare parametric variables such as logMAR and CSMT using the VassarStats statistical computation site [28]. The Fisher exact test was used to compare categorical outcomes. No correction was made for multiple comparisons and therefore all *p* values are nominal. A *p* value < 0.05 was considered statistically significant.

## 3. Results and Discussion

During the study period there were 234 patients who received IVT injections for DME. 192 eyes of 134 patients met inclusion criteria. There were 150 eyes in group 1 and 42 eyes in group 2. Demographic data for each group are shown in Table 1. Mean BCVA at baseline in group 1 was logMAR 0.47 (Snellen equivalent 20/59) and in group 2 was logMAR 0.55 (Snellen equivalent 20/70, *p* = 0.096). Mean CSMT at baseline was 424 *μ*m in group 1 and 429 *μ*m in group 2 (*p* = 0.43). There was an average number of 18 follow-up visits per patient in the review period.

A single type of anti-VEGF agent was used in 61 of the eyes in group 1 throughout the course of treatment while 73 eyes received 2 anti-VEGF agents and 16 were treated with all three anti-VEGF agents. Three eyes in group 2 received dexamethasone implants after a trial of triamcinolone acetonide was given. Of the patients in group 2 initially treated with anti-VEGF, 11 eyes (36.6%) received an IVT corticosteroid injection within 3 months and 20 eyes (66.6%) were treated with IVT corticosteroid within 6 months of initial anti-VEGF injection. Eyes in group 1 received fewer intravitreous injections compared to eyes in group 2 (6.9 versus 11.8 injections, *p* < 0.0001). Overall, 88.7% of eyes reviewed in this study received an injection of bevacizumab during the course of treatment.

### 3.1. Visual Acuity Outcomes

In group 1, there were 50% of patients with visual acuity of 20/40 or better at baseline, whereas in group 2 38% of patients presented with visual acuity of 20/40 or better (*p* = 0.22). At one year mean visual acuity improved to logMAR 0.41 (Snellen equivalent 20/51) in group 1 (*n* = 133) and worsened to logMAR 0.61 (Snellen equivalent 20/81) in group 2 (*p* = 0.003, Table 2). At final follow-up, mean visual acuity was logMAR 0.39 (Snellen equivalent 20/49) in group 1 (*n* = 150) compared to logMAR 0.61 (Snellen equivalent 20/84) in group 2 (*p* = 0.0005).

### 3.2. Anatomic Outcomes

At 1 year, CSMT in group 1 (*n* = 133) improved by −92 *μ*m compared to −46 *μ*m in group 2 (*p* = 0.049, Table 2). At final follow-up, CSMT in group 1 (*n* = 150) improved by −94 *μ*m compared to −68 *μ*m in group 2 (*p* = 0.26).

### 3.3. Subgroup Analyses

There were 23 eyes in group 2A (IVT-TA administered after at least 3 anti-VEGF injections without significant improvement in CSMT) and 19 eyes in group 2B (IVT-TA prior to receiving 3 anti-VEGF injections). As can be seen in Table 3, there were no significant differences between group 1, group 2A, and group 2B in initial CSMT or preinjection BCVA. Final CSMT was similar in the 3 groups (group 1: 330 *μ*m; group 2A: 340 *μ*m; group 2B: 357 *μ*m, *p* = 0.19). However, patients in group 1 had significantly better BCVA at final follow-up (group 1: 20/49; group 2A: 20/80; group 2B: 20/63, *p* = 0.0013). In contrast to eyes in group 1, eyes in subgroups 2A and 2B lost vision, on average (group 1: 4 letter gain; group 2A: 1 letter loss; group 2B: 6 letter loss, *p* = 0.035).

Only a minority of eyes were pseudophakic at the time of first injection (24 eyes in group 1 and 9 eyes in group 2). For this subgroup of eyes, mean visual acuity improved by 3.5 letters in group 1 compared to worsening by 2.5 letters in group 2 (*p* = 0.22). CSMT improved by −48 *μ*m in group 1 compared to an improvement of −64 *μ*m in group 2 (*p* = 0.71).

### 3.4. IOP Data

As shown in Table 4, at final follow-up, average IOP was stable in group 1 at 14.1 mmHg (*p* = 0.96) compared to baseline, whereas the mean IOP in group 2 had increased to 16.5 mmHg (*p* = 0.005). An increase in IOP by >10 mmHg was noted in 5 of 150 eyes (3.3%) in group 1 compared to 9 of 42 eyes (21%) in group 2 (*p* = 0.0005). Ocular antihypertensive drops were used in 4 of 150 eyes in group 1 compared to 8 of 42 eyes in group 2 (*p* = 0.0008), while 3 eyes underwent glaucoma surgery in group 2 compared to none in group 1 (*p* = 0.015).

### 3.5. Cataract Surgery

At final follow-up, 6 of 126 phakic eyes in group 1 compared to 12 of 33 phakic eyes in group 2 had undergone cataract surgery (4.7% versus 36.3%, *p* = 0.00009). Eyes that underwent cataract surgery in group 1 improved by a mean of 9 letters compared to eyes in group 2 that underwent cataract surgery that worsened by a mean of 13 letters (*p* = 0.05).

### 3.6. Discussion

In our study, IVT-TA used in combination with IVT anti-VEGF injections in eyes with center-involving DME led to worse visual and anatomic outcomes at 1 year compared to treatment with IVT anti-VEGF alone. Further, adjunct IVT-TA showed no benefit whether given early in the course of treatment or whether given to treat DME with incomplete response to 3 IVT injections of an anti-VEGF agent or agents. Worse, adjunct IVT-TA resulted in cataract progression in a third of our patients and ocular hypertension requiring treatment in a fifth of our patients. Previous studies have also shown a requirement for cataract surgery in up to 50% of patients and significant ocular hypertension in up to a third of patients [19]. Such cataract progression may have adversely affected visual outcomes; however, analysis of the small subgroups of eyes that were pseudophakic at baseline or eyes that underwent cataract surgery showed no benefit in visual or anatomic outcomes from adjunct IVT-TA.

Our results are consistent with those of previous publications comparing anti-VEGF to anti-VEGF with adjunct IVT triamcinolone acetonide for the treatment of DME [25–27, 29–31]. Ahmadieh and colleagues compared 3 monthly IVT bevacizumab injections to combination IVT bevacizumab with IVT-TA and reported no difference in visual and anatomic outcomes at 6 months [25]. Similarly, in another study, injections of IVT bevacizumab alone or in combination with IVT-TA were given at 12 weekly intervals if clinically significant DME was present and visual acuity was worse than 20/40: at 6 months visual and anatomic outcomes were similar [26]. Lim et al. [27] similarly found that while eyes treated with adjunct IVT-TA showed a more pronounced reduction in macular thickness in the early postinjection period, there was no beneficial effect in visual or anatomic outcomes with combination therapy at 1-year follow-up. In a study comparing bevacizumab with or without IVT-TA for refractory DME, TA seemed to induce earlier visual improvement, but no significant difference between CSMT or BCVA was found at 24 weeks [29]. Two studies, one with 90 patients and the other with 40 patients, found no significant difference in visual acuity at 3 months' follow-up in a single IVT bevacizumab injection compared to a single IVT bevacizumab injection combined with a single adjunctive IVT-TA injection [30, 31]. As intraocular VEGF levels have been correlated with the severity of DME, it is likely that consistent VEGF suppression with IVT anti-VEGF specific blockade yields a better anatomical result as seen in our study at one year [27, 32, 33]. A majority of patients in group 2 received IVT-TA only within the first year of treatment and this could explain why the anatomical success in group 1 was better at year 1. As patients received more IVT anti-VEGF throughout their treatment regimen the CSMT reduced more steadily and this is why we believe there was no statistically significant difference in anatomic outcomes at the end of the study period. While IVT-TA does suppress inflammatory mediators and vascular leakage, we hypothesize that the anti-VEGF suppression by IVT anti-VEGF agents has a more profound impact on DME.

When comparing all anti-VEGF therapies, a recent investigation found that aflibercept may be better than other intravitreal anti-VEGF medications when used in eyes with vision of 20/50 or worse [17]. Our investigation reviews patients receiving treatment for DME prior to this study's results and therefore the majority of our patients, regardless of initial visual acuity, received intravitreal bevacizumab at some point in their treatment regardless of visual acuity. It is possible that varying the treatment protocol based on which anti-VEGF is used may impact visual outcomes, and future prospective investigations should address these concerns.

Strengths of our study are that consecutive patients were included irrespective of severity of systemic diabetes or BCVA; the study had a longer follow-up and included a more ethnically diverse population (Caucasian, African-American, and Hispanic patients) unlike prior studies that included patients from the middle-East or Asian patients exclusively. Additionally, treatment was OCT-guided for center-involving DME as is current common practice in the US, with treatment being provided in a clinical setting and not in the rigid protocol of a randomized clinical trial. Limitations of the study were the retrospective study design (with the inherent risks for selection and treatment bias), an older and almost exclusively male population, with suboptimal glycemic control as evidenced by the baseline hemoglobin A1c values. It is possible that selection bias exists in this review, which can partially explain why the CSMT in the adjunct IVT-TA group did not improve as much as the IVT anti-VEGF only group. While no statistically significant difference existed in the baseline characteristics of the two groups, the group receiving adjunct IVT-TA may have had more chronic DME which could lead to bias in our results as these patients may have needed more treatment over time in order to obtain similar results as the IVT anti-VEGF only group. Further prospective randomized control trials are needed to limit the inherent risk of bias that exists in this study.

## 4. Conclusions

In this study of 192 eyes receiving IVT anti-VEGF treatment for center-involving DME, adjunct IVT-TA did not lead to improved visual or anatomic outcomes with follow-up of 1 year or beyond. This was true considering eyes that were pseudophakic at baseline or that underwent cataract extraction. Given the significant adverse events and the lack of support for benefit of being as an adjunct to anti-VEGF treatment for DME in this and previous studies, IVT-TA should be avoided unless patients have failed (worsening of CSMT or visual acuity) multiple IVT anti-VEGF agents (perhaps including IVT aflibercept given the protocol T results).

## Figures and Tables

**Table 1:** Baseline demographic and ocular data.

	Group 1	Group 2	*p* value
Patients (*N*)	107	27	—
Mean age, years	64.6	66.1	0.24
Male : female	106 : 1	27 : 0	0.99
White	59 (55.1%)	16 (59.2%)	0.33
African American	32 (29.9%)	6 (22.2%)	
Hispanic	6 (5.6%)	4 (14.8%)	
Other	10 (9.34%)	1 (3.70%)	
HbA1c	8.42%	8.17%	0.43
Mean follow-up	19.7 months	17 months	0.29
Eyes (*N*)	150	42	—
Phakic	126 (84%)	33 (79%)	0.46
Mean initial BCVA, logMAR	0.47 (20/59)	0.55 (20/70)	0.096
Mean initial CSMT	424 *μ*m	429 *μ*m	0.43
Mean initial IOP	14.2 mmHg	14.1 mmHg	0.91

**Table 2:** Main outcome results.

	Group 1	Group 2	*p* value
Mean 1-year BCVA, logMAR	0.41 (20/51)	0.61 (20/81)	0.003
Mean final BCVA, logMAR	0.39 (20/49)	0.63 (20/84)	0.0005
Average change in BCVA at 1 year	+2.5 letters	−2.5 letters	0.32
Average change in BCVA final	+3.5 letters	−3.5 letters	0.048
Mean CSMT at 1 year	332 *μ*m	381 *μ*m	0.039
Mean final CSMT	330 *μ*m	360 *μ*m	0.29
Average change in CSMT at 1 year	−92 *μ*m	−46 *μ*m	0.049
Average change in CSMT	−94 *μ*m	−68 *μ*m	0.26

**Table 3:** Subgroup analysis: outcomes in eyes that received early IVT-TA (group 2B) and eyes that received IVT-TA due to DME refractory to anti-VEGF treatment (group 2A).

	Group 1 (*n* = 150)	Group 2A (*n* = 23)	Group 2B (*n* = 19)	*p* value
Mean initial BCVA	0.47 (20/59)	0.58 (20/76)	0.51 (20/65)	0.25
Mean final BCVA	0.39 (20/49)	0.60 (20/80)	0.63 (20/85)	0.0013
Mean change in BCVA	+4 letters	−1 letter	−6 letters	0.035
Mean initial CSMT	424 *μ*m	440 *μ*m	391 *μ*m	0.20
Mean final CSMT	330 *μ*m	340 *μ*m	357 *μ*m	0.19
Mean change in CSMT	−94 *μ*m	−100 *μ*m	34 *μ*m	0.28

**Table 4:** Ocular adverse events.

	Group 1	Group 2	*p* value
Mean initial IOP	14.2 mmHg	14.1 mmHg	0.93
Mean final IOP	14.1 mmHg	16.5 mmHg	0.0005
Increase > 10 mmHg from baseline	5 (3.3%)	9 (21.4%)	0.0005
Initiation of IOP-lowering medication at any visit	4 (2.7%)	8 (19.0%)	0.0008
Glaucoma surgery	0	3 (7.1%)	0.015
Cataract surgery	6 (4.7%)	12 (36.3%)	0.0009
